# Anterior cruciate ligament reconstruction-related patient injuries: a nationwide registry study in Finland

**DOI:** 10.1080/17453674.2019.1678233

**Published:** 2019-10-15

**Authors:** Kirsi-Maaria Nyrhinen, Ville Bister, Teemu Helkamaa, Arne Schlenzka, Henrik Sandelin, Jerker Sandelin, Arsi Harilainen

**Affiliations:** a Department of Orthopaedics and Traumatology, Helsinki University Central Hospital;; b Department of Surgery, Hyvinkää Hospital, Hyvinkää;;; c Orthopaedic Department, Liverpool Hospital, Sidney, New South Wales, Australia;; d ORTON Orthopaedic Hospital, Invalid Foundation, Helsinki, Finland

## Abstract

Background and purpose — Treatment outcomes of anterior cruciate ligament (ACL) injuries are generally good, but complications after ACL reconstruction (ACLR) can result in long-lasting problems. Patient injury claims usually fall on the more severe end of the complication spectrum. They are important to investigate because they may reveal the root causes of adverse events, which are often similar regardless of the complication’s severity. Therefore, we analyzed ACL-related patient injuries in Finland, the reasons for these claims, causes of complications, and grounds for compensation.

Patients and methods — We analyzed all claims filed at the Patient Insurance Centre (PIC) between 2005 and 2013 in which the suspected patient injury occurred between 2005 and 2010. This study also reviewed all original patient records and available imaging studies. General background data were obtained from the National Care Register for Social Welfare and Health Care (HILMO).

Results — There were 248 patient injury claims, and 100 of these were compensated. Compensated claims were divided into 4 main categories: skill-based errors (n = 46), infections (n = 34), knowledge-based errors (n = 6), and others (n = 14). Of the compensated skill-based errors, 34 involved graft malposition, 26 of them involved the femoral-side tunnel. All compensated infections were deep surgical site infections (DSSI).

Interpretation — This is the first nationwide study of patient injuries concerning ACLRs in Finland. The most common reasons for compensation were DSSI and malposition of the drill tunnel. Therefore, it would be possible to decrease the number of serious complications by concentrating on infection prevention and optimal surgical technique.

Anterior cruciate ligament reconstruction (ACLR) has a high success rate, and most patients can return to sporting activities postoperatively with nearly normal knee function. Complications are rare but can be devastating. A new trauma, technical errors, graft failure, problems with the fixation methods, postoperative infections, and venous thrombosis are the most common complications and often lead to reoperations and prolonged rehabilitation (Schulz et al. 2007, Saper et al. 2014, Magnussen et al. 2015, Christensen and Miller 2018). However, the literature contains little information on ACLR-related patient injury claims.

A range of patient injuries in Finland have been previously investigated by several research groups, including total hip and knee arthroplasty, children’s tibial and femoral fractures, distal radius fractures, and fatal complications (Palmu et al. 2009, 2010, Järvelin et al. 2012, Hakala et al. 2014, Helkamaa et al. 2016, Sandelin et al. 2018). This study analyzes and describes ACL-related patient injuries in Finland and the root causes that lead to these complications.

## Patients and methods

This is a descriptive retrospective register study. Unlike other Nordic countries, there is no national anterior cruciate ligament (ACL) register in Finland. However, there is the Care Register for Social Welfare and Health Care (HILMO), which is maintained by the National Institute for Health and Welfare (THL). The HILMO collects national information from all healthcare sectors on patients who undergo an operation. This study obtained background data on ACL patients treated in Finland between 2005 and 2010 from the HILMO. Data on claims and claimants was obtained from the Patient Insurance Centre (PIC; www.pvk.fi/en/). ACL injuries were identified from this national register through the International Classification of Diseases 10th edition (ICD-10) codes (Table 1, see Supplementary data), and ACLRs were identified through the Nordic Medico-Statistical Committee (NOMESCO) procedure codes NGE30 and NGE35. The code NGE30 stands for “open ACLR,” and NGE35 stands for “arthroscopic ACLR.” The study period 2005 to 2010 was chosen to allow for the complete processing of all patient injury claims by the PIC. It can take 1 to 7 years to collect the necessary material, analyze it, and sometimes even re-analyze it. All patient injury claims obtained for this study were related to the ICD-10 code S83.5. They were analyzed by reviewing the claimants’ original patient records and imaging studies (when available).

**Table 2.** Criteria entitling patient to compensation according to the Finnish Patient Injury Act (Patient Injury Act 25.7.1986/585)

1.	Treatment injury
2.	Equipment-related injury
3.	Infection injury
4.	Accidental injury
5.	Injury from damage to healthcare facilities
6.	Injury due to delivery of pharmaceuticals
7.	Unreasonable injury

The PIC collects statistics and promotes research to support patient health and safety. Public and private healthcare units must have patient injury insurance. The insurance companies that offer this insurance must be members of the PIC. A single healthcare unit or a healthcare district cannot be a member of the PIC directly. Patient injuries have been centralized to the PIC in Finland. The PIC’s function is based on the Patient Injury Act. According to the Act, there are 7 criteria that entitle a patient to compensation (Table 2). At least 1 of the 7 criteria must be met to comprise a patient injury case (Patient Injury Act 1986). Finland and the other Nordic countries have a no-fault patient insurance system as opposed to the tort insurance system in the United Kingdom and United States (Palonen et al. 2005, Järvelin and Häkkinen 2012, Patient Insurance Centre n.d.). If a patient is unsatisfied with their care for any reason, the PIC offers an impartial estimation concerning that care. When the claim arrives at the PIC, all information regarding the care in question is collected. Using this information, an independent specialist estimates whether an experienced healthcare professional would have treated the patient differently and if, thereby, the event leading to the compensation claim could have been avoided. When infection or unreasonable injury is suspected, a specialist and the PIC evaluate whether the consequences of the complication are too much for the patient to tolerate. Therefore, the operation of the PIC is based on the following questions: was the injury preventable and was the consequence tolerated (tolerable disadvantage, temporary disadvantage, or permanent dis­advantage) (Mikkonen 2004, Helkamaa et al. 2016, Patient Insurance Centre n.d.). In general, patients have 3 years to file a claim after treatment. If the claimant is not satisfied with the decision, he/she may refer the claim to the Patient Injury Board, which consists of several independent specialists. If the claimant is still dissatisfied, he/she can take the claim to the general court. This rarely occurs.

The PIC compensates the true expenses that are due to the patient injury. These include the cost of hospital care, visits to outpatient clinics, rehabilitation, visiting nurse services, and laboratory and imaging studies. If there is a decline in work performance or loss of primary income, an estimation of work ability is made. In these cases, compensation is paid from the patient’s insurance to prevent a decline in income. In addition, the PIC defines the disability and harm that results from the patient’s injury as either transient or permanent and compensates this accordingly. The severity of the patient’s injury, their recovery time, their level of required care, and any patient-specific factors all affect the decision and amount of compensation (Patient Insurance Centre n.d.).

### Ethics, funding, and potential conflicts of interest

Permission for this study was provided by the PIC and the Ethical Board of Helsinki University (376/13/03/02/2015). No funding was received. The authors have no conflicts of interest.

## Results

### Background data: HILMO

**Figure 1.**
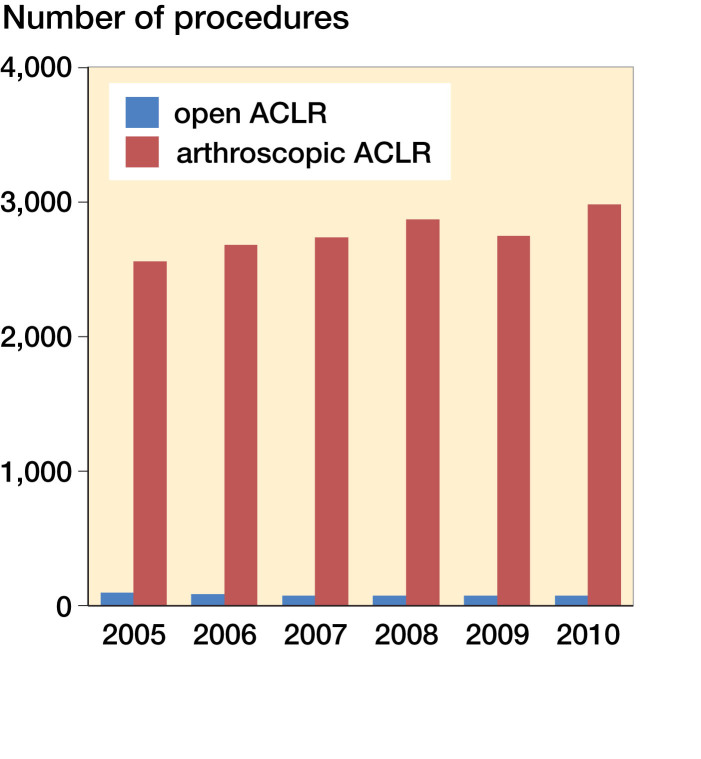
ACL reconstructions or revisions (n) (procedure codes NGE30 and NGE35) between 2005 and 2010.

**Figure 2.**
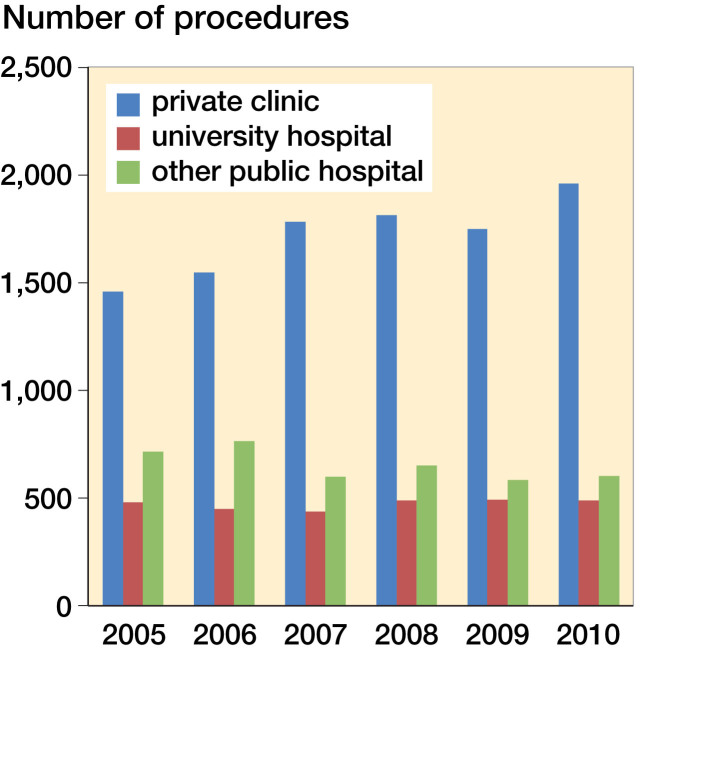
Distribution of different healthcare units performing ACL surgery between 2005 and 2010 in Finland.

Between 2005 and 2010, 31,643 patients (64% men) were diagnosed with an ACL injury (S83.5). During the same period, 17,041 ACLRs or revision procedures were performed in Finland (Figure 1). It is impossible to separate revisions from primary reconstructions based on registry data alone because the NOMESCO procedure codes are the same. The distribution of healthcare units is provided in Figure 2. The distribution of sex and the mean age of ACLR patients within the HILMO data and patient injury claimants in the PIC data are provided in Table 3.

**Table 3.** Total number, sex distribution, and mean age of anterior cruciate ligament reconstruction (ACLR) patients (HILMO) and patient injury claimants (PIC) between 2005 and 2010: background data from the HILMO and research data from the PIC

Sex	HILMO: ACLR patients		PIC: patient injury claims	
	n	mean age (range)	n	mean age (range)
Male	11,293	31 (4–76)	141	35 (9–71)
Female	5,646	35 (6–77)	107	35 (8–75)
Total	16,939	33 (4–77)	248	35 (8–75)

**Table 4.** Reasons for patient complaints^a^

Reason	n
Pain	95
Financial difficulties (sick leave, unemployment, additional expenses)	60
Infection	56
Reoperation	53
Decline in work performance	48
Delay in care	45
Delay in diagnosis	38
Decline in general performance	31
Prolonged rehabilitation	29
Prolonged use of antibiotics	22
Edema	15
Instability	15
Numbness	13
Arthrosis	11
Deep venous thrombosis	10

a Patients complained for 65 different experienced reasons. Each of those reasons have been collected and are presented in this table. Reasons that appeared less than 10 times are not presented.

### The research data: PIC

This study found 248 filed patient injury claims between 2005 and 2013 that concerned a suspected ACL-related patient injury occurring between 2005 and 2010. Though injuries generally occurred during sports (n = 117), 67 occurred during leisure time and 41 were work-related. The remaining 23 were traffic accidents, accidents at home, or occurred under circumstances that remain unclear. 239 injuries were treated operatively. Of these, 231 were ACLRs, 2 were ACL avulsion fixations, and 6 were other surgical procedures. All ACLRs, compensated or not, were performed with arthroscopic assistance. A hamstring graft was used in 188 of these operations, a bone–tendon–bone graft (BTB) was used in 33, and different graft sources were used in the remaining cases. An anteromedial (AM) drilling technique was used in 111 operations, a transtibial (TT) drilling technique was used in 74, and it was impossible to confirm the drilling technique used in 45 as the data were missing or the surgery reports were incomplete. The average operation time was 72 min (33–191). Use of a prophylactic antibiotic was documented in 196 operations, most commonly a single dose of intravenous cefuroxime 1.5g (n = 137).

### Reasons to file a claim (Table 4)

**Figure 3.**
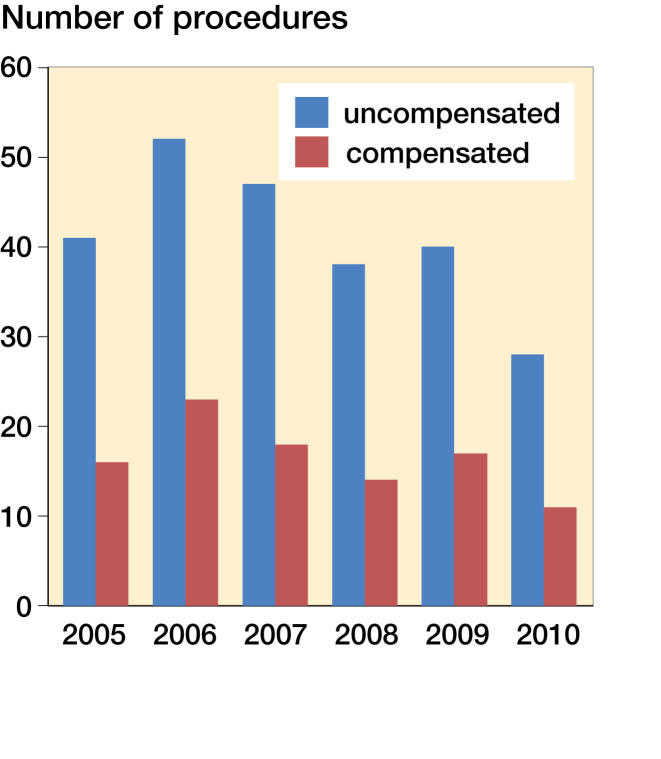
Patient injury claims during 2005–2010.

Figure 3 shows patient injury claims (n = 248) between 2005 and 2010 based on the search criteria. On average, 41 ACLR-related patient injury claims were filed annually, and 17 claims were compensated.

### Compensated claims

The PIC compensated 100/248 of all ACLR-related claims (Table 5, see Supplementary data). The 2 most common reasons for compensation were technical errors/skill-based errors (n = 46) and postoperative deep surgical site infections (DSSI) (n = 34). Only 6 claims were reimbursed due to knowledge-based errors. The remaining claims were single cases. The average age of the compensated claimants at the time of their injury was 33 years (14–75), and 55 of them were male.

### Skill-based errors (n = 46)

Technical errors leading to instability, loss in a patient’s range of motion (ROM), loss of a graft, or reoperation are considered patient injuries. Of all compensated patient injury claims, 34/100 were due to graft malposition. The most common error was to the anterior femoral tunnel, which was compensated in 15 cases (Table 6, see Supplementary data).

In 9 cases, an additional arthroscopy was performed. This occurred when a ruptured ACL was not reconstructed during the first arthroscopy (n = 5) or a broken instrument or fixation material needed to be removed after ACLR (n = 4).

There were 6 claims concerning saphenous nerve problems after ACLR but only 2 were compensated.

In 1 case, the BTB graft was damaged at the bone–ligament junction. It was estimated to be long enough without the bone block and was fixed with a screw to the femoral canal. During follow-up, the knee became loose and required a revision. Compensation was granted because, with a careful operation technique, the BTB graft would have remained intact and a revision could have been avoided.

### Infections (n = 34)

All compensated infections were DSSI, and they comprised 34/100 of the compensated claims. The graft was lost in 20/34 of these infections. There was a mean of 1.6 (1–6) arthroscopic lavages per infection. The treatment period at the hospital after infection was on average 14 days (2–33). The most common bacteria were Staphylococcus aureus (n = 9), coagulase-negative coccus (n = 4), and Staphylococcus epidermidis (n = 3). In 13 infection cases, the bacteria could not be identified, despite the culture, or the information was missing. A hamstring graft was used in 30/34 cases, a BTB graft was used in 2/34 cases, and the surgery report was missing in 2/34 cases. A prophylactic antibiotic was used in 27/34 of the postoperative infections, while 3/34 did not receive any antibiotics before their operation. In 4/34 cases, information on prophylactic antibiotics was missing.

### Paid compensations

The PIC paid €823,800 in compensation to 93 patients. In 7 of these cases, the documents concerning the paid compensations were missing. The median value per patient injury was €5,600 (€400–126,000). The largest compensation was for an infection that led to a 2-phase reconstruction. The minimum amount was paid to 3 patients who were considered to have suffered only transient discomfort due to additional arthroscopy (Table 7, see Supplementary data).

### Comparison of HILMO and PIC register data

In a comparison of the total number of ACLRs (17,041) to the PIC materials containing 231 ACLRs, only 1.4% of ACLRs were sent to the PIC. There were 34 compensated DSSIs among the 17,041 ACLRs. According to these data, the risk of a DSSI severe enough to result in compensation was 0.2%. Furthermore, there were 56 infection-related claims. Therefore, the overall risk of infection was at least 0.3%.

## Discussion

This is the first nationwide study of ACL-related patient injury claims and compensation in Finland. Filed patient injuries usually entail more severe complications because these are more readily reported. These claims are important to investigate because they can reveal the root causes of complications that can often be similar regardless of severity. The most common grounds for compensation found were technical errors and infections.

This study’s results agree with the current knowledge regarding ACLR-related complications. The femoral side is the most difficult side on which to drill and errors are, therefore, most likely to occur there (Sommer et al. 2000, Wright et al. 2010, Morgan et al. 2012, Chen et al. 2013). Most compensated graft malpositions (26/34) were due to a technical error on the femoral side. The length and tension of the graft is determined by the positioning of the femoral drill tunnel. There are criteria that guide the positions of both the femoral and the tibial drill tunnels (Bernard et al. 1997, Marchant et al. 2010, Kopf et al. 2012, Robin and Lubowitz 2014, Samitier et al. 2015, Robin et al. 2015). This study’s findings suggest that, simply by following existing standard surgical techniques, many ACLR-related patient injuries could be avoided.

Infections were the second most common reason for compensation in this study. The incidence of infection after ACLR is 0.5–1%, according to contemporary literature (Westermann et al. 2017, Bansal et al. 2018). Postoperative infections can cause severe, long-lasting consequences. Antibiotics are used for several weeks, and the infected knee often requires arthroscopic lavages. Eventually, the graft is often lost and a 1- or 2-phase revision is needed. In the worst scenario, the infection leads to poor joint function and arthrosis (Vertullo et al. 2012). Compensation is granted if the postoperative infection is unexpected (the patient has no predisposing health conditions that push the infection risk above 2%) and the consequences are so severe that patient should not have to tolerate them. There were more infections among patients whose operations included hamstring grafts, as previously reported (Maletis et al. 2013, Gifstad et al. 2014, Okoroha et al. 2016, Westermann et al. 2017, Randsborg et al. 2018). Among the compensated claims, a hamstring graft was the most common. However, the exact percentage of hamstring grafts used in Finland during ACLRs is unknown. In Norway, this has led to an increased use of BTB grafts. However, Pérez-Prieto et al. (2016) and Phegan et al. (2016) have found that the likelihood of postoperative infection can be significantly reduced if the hamstring graft is pre-soaked in a vancomycin solution.

In 2018, Randsborg et al. published their study on 101 compensated claims as a result of ACLRs performed in Norway from 2005 to 2015. The most common reason for compensation was postoperative infection (39%), the second most common was inappropriate surgical technique (27%), and the third most common was delayed diagnosis (13%). Risk of postoperative infection increased when a hamstring graft was used. The Norwegian register reveals that the use of hamstring grafts decreased from 2005 to 2015. The reason for this is the higher risk of revision. This same trend was previously demonstrated by Persson et al. (2014) who published their results regarding the Norwegian Cruciate Ligament Registry between 2004 and 2012. In addition, technical errors, and especially an incorrect femoral tunnel placement, were common mistakes. These findings correspond with this study’s findings. Functional results between the BTB graft and the hamstring graft are similar despite the anterior knee pain, which is more common with a BTB graft. Risk of infection and risk of a graft failure increase with a hamstring graft (Gifstad et al. 2014, Okoroha et al. 2016).

Other patient injuries detected in this study included diagnostic errors and treatment delays, which were clinically relevant errors. After an injury, patients often visit an emergency department or a general practitioner who does not have access to magnetic resonance imaging (MRI). A clinical diagnosis of an ACL rupture is difficult in an acute phase, and it is therefore difficult to estimate an acceptable delay. According to contemporary literature, ACL reconstruction should be performed within 1 to 6 months following the injury (Francis et al. 2001, Taketomi et al. 2018). However, determining a diagnosis is difficult and recommendations for scheduling surgery vary. Therefore, it can be challenging to estimate whether a patient has suffered too much because of a delay and a patient injury has occurred.

This study has 2 major limitations. Due to the retrospective study design, this study suffers from the limitations of the registry. Finland does not have a national ACL register; therefore, it is difficult to determine the operation volumes of healthcare units or single surgeons. Furthermore, this study is unable to separate primary ACLRs from revisions or to assess the exact number of ACL patients because the ICD-10 codes do not distinguish between ACL and PCL injuries. In addition, the exact information on grafts used, fixation methods, drilling techniques, and rehabilitation programs at the national level remain unknown. To compensate for these shortcomings, this study combined 2 nationwide databases and analyzed the original patient records and imaging studies (when available) of all patient injuries. This allowed the study to gather more specific data and exclude any errors that commonly occur when only registry data are used. As previous studies have demonstrated, the coverage and accuracy of the HILMO is very good (Mattila et al. 2008, Sund 2012).

Patients do not complain as often concerning mild complications as they do for severe complications. Although reported patient injuries usually fall on the more severe end of the complication spectrum, they can still be investigated to determine a wide range of complications. The root causes of adverse events are often similar regardless of the complication’s severity. Based on these data, this study calculated that the overall risk of infection after ACLR in Finland was at least 0.3%. Based on the average risk of infection after ACLR in the literature (Westermann et al. 2017, Bansal et al. 2018), this study can estimate that coverage of serious complications, such as infection, is fairly good according to PIC data. The rate of coverage may not be 100%, but it is far better than what was previously suggested by Pukk et al. (2003) who argued that patient injury claims would represent only 3% of patients who had a complication that would fulfill patient injury criteria. Therefore, the conclusions drawn from this registry study are more likely to be widely applicable and reliable. In addition, this study’s results regarding typical error types are in line with previous studies regarding Scandinavian ACL registry data (Persson et al. 2014, Randsborg et al. 2018).

## Conclusion

Complications leading to filed patient injury claims are quite rare after ACL reconstructions, but they can lead to devastating consequences. According to this study, the best way to reduce ACLR-associated complications is the prevention of DSSIs, optimal femoral canal drilling, and optimal graft placement.

## Supplementary Material

Supplemental Material

## Supplementary data

Tables 1, 5, 6, and 7 are available as supplementary data in the online version of this article, http://dx.doi.org/10.1080/17453674.2019.1678233
