# Supplementary material for: Anterior cruciate ligament reconstruction-related patient injuries: a nationwide registry study in Finland
Source: Acta Orthop. 2019 Oct 15;90(6):596–601. doi: 10.1080/17453674.2019.1678233 (PMC6844426; doi:10.1080/17453674.2019.1678233)
Supplement: Supplemental Material [file IORT_A_1678233_SM6659.pdf]

Supplementary data

Table 1. The International Classification of Diseases 10th edition (ICD-10) codes used as search criteria at the PIC

ICD10-code	
S83.5	Sprain of the cruciate ligament(s) of the knee
S83.6	Sprain and strain of other and unspecified parts of the knee
S83.7	Injury of multiple structures of the knee
S89.7	Multiple injuries of the lower leg
S89.9	Unspecified injury of the lower leg
M23.5	Chronic instability of the knee
M23.6	Other spontaneous disruption of ligament(s) of the knee
M23.8	Other internal derangements of the knee
M24.2	Disorder of a ligament

Table 5. Compensated claims. Values are frequency

Skill-based errors (n = 46)	
Graft malposition	34
ACLR not done during primary arthroscopy	5
Removal of fixation material or broken drill bit	4
Saphenous nerve lesion	2
Broken BTB graft was used	1
Infections (n = 34)	
Deep infection	34
Knowledge-based errors (n = 6)	
Problems of healthcare organization	3
Delay in primary diagnosis	3
Other (n = 14)	
Failed osteotomy before ACLR	3
Antithrombotic dose too high	2
No antithrombotic used in high-risk patient	2
Anesthesiological problem	1
Re-rupture during postoperative patient transfer	1
Wrong knee was operated on	1
Multifactorial reasons	4

Table 6. Errors when drilling the tunnels. Values are frequency

Error type	TT	AM	Unknown	Total	Femoral side
Femoral tunnel too anterior	10	5		15	15
Femoral tunnel too vertical	3	1	1	5	5
Tibial tunnel too posterior	2	3		5	
Femoral tunnel too posterior, blow out		3		3	3
Graft position too anterior			1	1	
Both tunnels too anterior	1			1	1
Tibial tunnel too vertical	1			1	
Tibial tunnel too anterior	1			1	
Femoral tunnel not optimal [a]		1		1	1
All tunnels suspicious [b]			1	1	1
Total				34	26

[a] No specific argument.
[b] After ACLR and revision it was difficult to estimate.
TT = transtibial drilling technique, AM = anteromedial drilling technique, unknown = unknown drilling technique.

Table 7. Paid compensations in €

Skill-based errors (n = 46)	
Graft malposition	280,100 [a]
ACLR not done during primary arthroscopy	13,700
Removal of fixation material or broken drill bit	25,800
Saphenous nerve lesion	16,400
Broken BTB graft was used	6,800
Infections (n = 34)	
Deep infection	420,200 [b]
Knowledge-based errors (n = 6)	
Problems of healthcare organization and delay in primary diagnosis	19,200 [c]
Other (n = 14)	
Failed osteotomy before ACLR	23,800
Antithrombotic dose too high	4,400 [c]
No antithrombotic used in high-risk patient	2,600
Anesthesiological problem	900
Re-rupture during postoperative patient transfer	600
Wrong knee was operated on	2,900
Multifactorial reasons	6,300

[a] In 3 cases, documents concerning the paid compensation missing.
[b] In 2 cases, documents concerning the paid compensation missing.
[c] In 1 case, documents concerning the paid compensation missing.
